# Selective medium for culture of *Mycoplasma hyopneumoniae*

**DOI:** 10.1016/j.vetmic.2016.09.022

**Published:** 2016-11-15

**Authors:** Beth S. Cook, Jessica G. Beddow, Lucía Manso-Silván, Gareth A. Maglennon, Andrew N. Rycroft

**Affiliations:** Department of Pathology & Pathogen Biology, Royal Veterinary College, Hawkshead Lane, North Mymms, AL9 7TA, UK

**Keywords:** *Mycoplasma hyopneumoniae*, Selective culture, Enzootic pneumonia, Pig

## Abstract

The fastidious porcine respiratory pathogen *Mycoplasma hyopneumoniae* has proven difficult to culture since it was first isolated in 1965. A reliable solid medium has been particularly challenging. Moreover, clinical and pathological samples often contain the fast-growing *M. hyorhinis* which contaminates and overgrows *M. hyopneumoniae* in primary culture. The aim of this study was to optimise the culture medium for recovery of *M. hyopneumoniae* and to devise a medium for selection of *M. hyopneumoniae* from clinical samples also containing *M. hyorhinis*. The solid medium devised by Niels Friis was improved by use of Purified agar and incorporation of DEAE-dextran. Addition of glucose or neutralization of acidity in liquid medium with NaOH did not improve the final yield of viable organisms or alter the timing of peak viability. Analysis of the relative susceptibility of *M. hyopneumoniae* and *M. hyorhinis* strains to four antimicrobials showed that *M. hyopneumoniae* is less susceptible than *M. hyorhinis* to kanamycin. This was consistent in all UK and Danish strains tested. A concentration of 2 μg/ml of kanamycin selectively inhibited the growth of all *M. hyorhinis* tested, while *M. hyopneumoniae* was able to grow. This forms the basis of an effective selective culture medium for *M*. *hyopneumoniae*.

## Highlights

• Systematic improvements to the medium for growth of *Mycoplasma hyopneumoniae*.
• Development of selective medium for *M. hyopneumoniae* inhibitory to *M. hyorhinis*.
• Improved colony size of *M. hyopneumoniae*.
• Improved reproducibility of culture for use in genetic manipulations.

## 1 Introduction

*Mycoplasma hyopneumoniae* is the primary cause of enzootic pneumonia (EP) in pigs. The disease is of global significance both in reducing growth efficiency and in promoting susceptibility to concurrent bacterial and viral infections (Thacker et al., 1999). Diagnosis of EP is usually by a combination of serological tests (Feld et al., 1992, Djordjevic et al., 1994), PCR of nasal secretion or lung tissue at slaughter (Mattsson et al., 1995, Baumeister et al., 1998, Stemke, 1997), real-time PCR (Dubosson et al., 2004, Marois et al., 2010) and isolation in culture (Kobisch and Friis, 1996). However, *M. hyopneumoniae* is notoriously fastidious (Goodwin and Hurrell, 1970) and culture remains challenging and time consuming. The most widely used liquid medium for culture of *M. hyopneumoniae* was developed by Niels Friis (1975). Homogenised lung tissue was serially diluted in tubes of the medium and incubation led to a gradual colour change of the phenol red indicator from pink to yellow, without turbidity, over a period of 3–10 days growth. However, individual mycoplasmas could only be purified by terminal dilution in the liquid medium.

A commercial medium is available from Mycoplasma Experience^®^ Ltd. U.K. (ME). Both liquid and solid media are available. These support the growth of *M. hyopneumoniae* but the constituents of this commercial medium are not publicly available. Solidification of Friis medium with Agar did not allow growth of colonies of *M. hyopneumoniae*, suggesting that the agar was inhibitory to growth or sequestered an essential nutrient. The use of a better defined solid medium would allow greater flexibility for research, such as transformation of *M*. *hyopneumoniae* (Maglennon et al., 2013a).

When used for diagnostic purposes, both Friis medium and ME media are easily overgrown by the faster-growing *M. hyorhinis* which is sometimes present in pig lung lesions alongside *M. hyopneumoniae* and frequently in apparently normal tissue (Kobisch and Friis, 1996). To suppress the growth of *M. hyorhinis*, Friis recommended the use of 5% hyperimmune anti-*M. hyorhinis* rabbit serum and 500 μg/ml cycloserine (Friis, 1971). However, such serum is not always available, is expensive, and batches of serum vary in their capacity to suppress *M. hyorhinis*.

The purpose of this study was therefore to improve the culture medium for transformation and mutagenesis of *M. hyopneumoniae*, allowing single colonies to be readily distinguished and selected, and to devise an improved selection medium for the suppression of *M. hyorhinis*.

## 2 Materials & methods

### 2.1 Mycoplasma strains

*M. hyopneumoniae* strains 277/94 and 325/95 were the gift of Niels Friis, Danish Veterinary Institute, Copenhagen. Other strains of *M. hyopneumoniae* were Danish field isolates from lesions of EP, the gift of Dr Branko Kokotovic, Danish Veterinary Institute, or UK field isolates from slaughterhouse lesions of EP. *M. hyorhinis* strains were UK field isolates obtained from pig lungs with or without gross lesions at post-mortem. The identity of all organisms was confirmed using species-specific PCR amplifying a region of the conserved hypothetical protein mhp165 from *M. hyopneumoniae* (Baumeister et al., 1998) and the highly conserved 16S rRNA region of *M. hyorhinis* (Lin et al., 2006). The identity of all *M. hyopneumoniae* strains was subsequently confirmed by whole genome sequencing and genome-wide analysis (J. Welch − personal communication).

### 2.2 Liquid culture medium

Friis medium was prepared largely as described by Kobisch and Friis (1996) with the following modifications: bacitracin and meticillin were replaced with azlocillin and flucloxacillin (final concentration 50 μg/ml). To make 500 ml of Friis medium, 1.5 g Brain Heart Infusion (BHI) (Difco) and 1.6 g PPLO (Difco) was dissolved in 365 ml water and sterilised by autoclaving. To this were added 18 ml of yeast extract (prepared from dried bakers yeast), 12.5 ml sterile solution A (160 g/l NaCl, 4 g/l; 8 g/l KCl, 2 g/l MgSO_4_·7H2O, 2 g/l MgCl_2._6H_2_O, 3.7 g/l CaCl_2_·2H_2_O), 12.5 ml sterile solution B (3.0 g/l Na_2_HPO_4_.12H_2_O, 1.2 g/l KH_2_PO_4_), 50 ml pig serum (Invitrogen) heat-treated at 56 °C for 20 min, 50 ml heat-treated horse serum (Invitrogen), 1 ml phenol red solution (0.6% in 0.1 M NaOH), 25 μl of each of 100 mg/ml azlocillin and flucloxacillin and adjusted to pH 7.4 with 1.0 M NaOH.

### 2.3 Solid culture medium

Concentrated (2.8×) Friis medium was prepared by the addition of 88 ml water to 4.3 g BHI, 4.6 g PPLO sterilised by autoclaving, 51.4 ml yeast extract, 35.7 ml sterile solution A, 35.7 ml sterile solution B, 143 ml heat-treated horse serum, 143 ml heat-treated pig serum, 2.8 ml 0.6% phenol red solution, azlocillin and flucloxacillin (final concentration 140 μg/ml), pH 7.4. Friis agar was prepared by mixing concentrated Friis medium (2.8×), mixed at a ratio of 35: 65 with either 1.8% agar No. 1 (Oxoid, L11), Purified agar (Oxoid, L28) or agarose (Invitrogen). Where indicated, DEAE-dextran (Sigma-Aldrich, Gillingham, UK) was added to agar at 0.1 mg/ml. Mycoplasma Experience^®^ solid medium was prepared according to the manufacturer’s instructions (Mycoplasma Experience^®^ Ltd, Reigate, UK). Cultures were incubated in a humidified incubator at 37 °C in 5% CO_2_.

Charcoal-treated agar was prepared by incubating a 1.3% solution of Bacteriological agar with 2% w/v of activated charcoal (Oxoid, Basingstoke, UK) for 10 min at 55° C, the charcoal was then removed using a sieve. Following sterilisation, each agar type was mixed with medium supplement (Mycoplasma Experience Ltd, UK) or concentrated Friis medium at a ratio of 35: 65 (supplement: agar) and plates poured.

### 2.4 Viability determination

Ten-fold serial dilutions in Hanks’ Balanced salts Solution (HBSS; Life Technologies) were spotted (10 μl in triplicate) on to solid medium. Cultures were incubated for 7 days in 5% CO_2_ at 37 °C. Colonies were counted, from those dilutions showing the largest number of separate colonies, using a dissecting microscope; the triplicate counts were combined and the viability calculated. Growth was also estimated from the metabolic activity recorded as a colour shift of the phenol red pH indicator from pH 7.4 to 6.8 as described by Friis (1975).

### 2.5 Antibiotic susceptibility testing

Solutions of kanamycin (Sigma), tetracycline (Sigma) and puromycin (Fisher) were prepared in de-ionised water. Chloramphenicol (Sigma) was dissolved in ethanol at 10 mg/ml final concentration. All were sterilised through a 0.2 μm filter (Nalgene, UK). A final antibiotic concentration range was achieved by two-fold serial dilutions of each antibiotic in sterile water or ethanol as appropriate. Antibiotics were incorporated into solid medium over a range of concentrations 20–0.01 μg/ml (kanamycin), 4.0–0.03 μg/ml (tetracycline), 20–0.06 μg/ml (chloramphenicol and puromycin)

*M. hyopneumoniae* and *M. hyorhinis* inocula were prepared in 1 x sterile phosphate buffered saline (BR0014G, Oxoid) as a suspension containing approximately 10^3^ CFU/ml. A sample of 10 μl of each strain was inoculated onto each plate in triplicate. Plates were incubated at 37° C with 5% CO_2_ and observed for growth of colonies between day 2 and day 10. The MIC was determined as the highest dilution of antibiotic giving an absence of growth as determined by the formation of mycoplasma colonies.

## 3 Results

### 3.1 The relationship of viability and colour shift

**Fig. 1:**
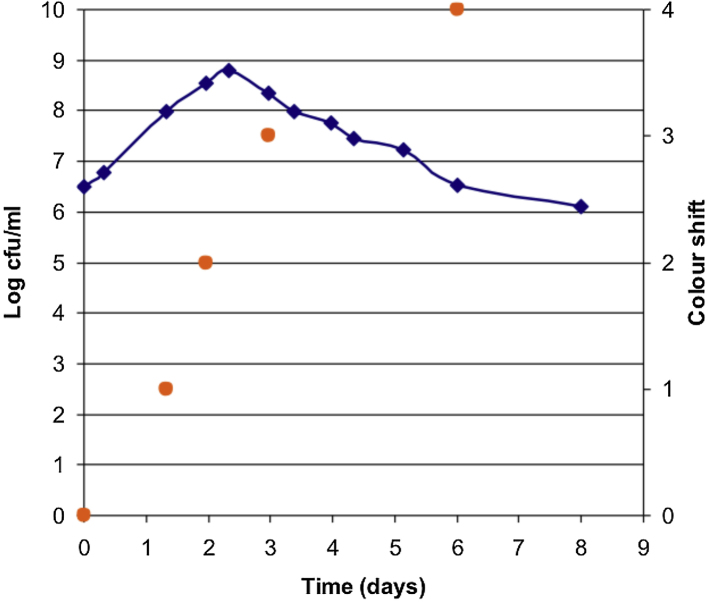
Viability and colour shift of *M. hyopneumoniae* cultured in Friis broth. Viable cell counts were performed every 12 h and the colour shift recorded. Results are from two independent determinations of the growth curve.

The viability of *M. hyopneumoniae* to the commonly used colour shift in liquid medium was determined (Fig. 1). Cultures reached a maximum viability of 10^8.9^ at approximately 2.5 days incubation. This corresponded to a colour shift between 2 (pH 7.2) and 3 (pH 7.1). After this point there was an exponential decline reaching 10^6^ CFU/ml after 8 days (beyond colour shift 4).

### 3.2 The effect of glucose and NaOH

**Fig. 2:**
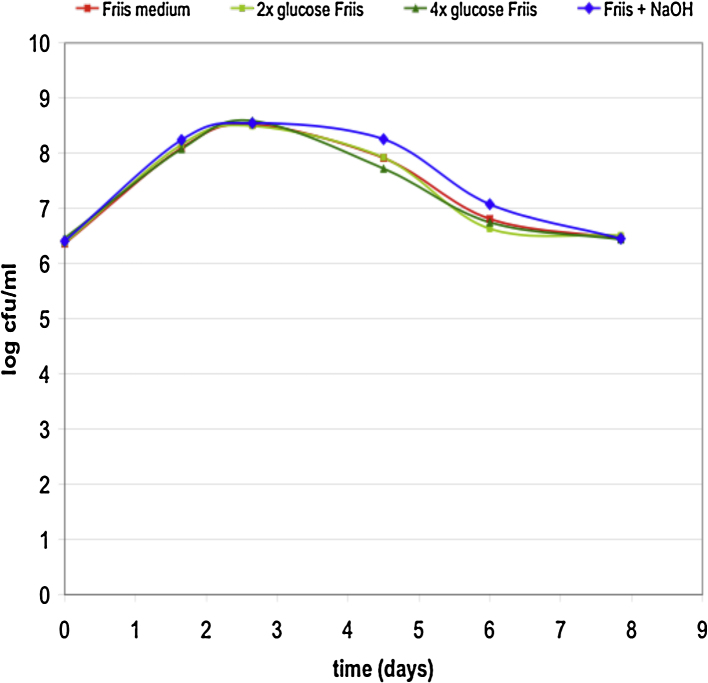
Effect of glucose and alkali on the growth curve of *M. hyopneumoniae*. Viable counts were performed at intervals. Friis broth contained either ×2 or ×4 the normal concentration of glucose. The culture (NaOH) was neutralized to pH 7.3 at 2.5 days post inoculation.

The concentration of glucose in Friis medium is approximately 2 mM: 1 mM is derived from the brain heart infusion broth and another 1 mM from the diluted serum. To investigate whether glucose is limiting the growth, further glucose was supplemented into the liquid medium to give 4 mM (×2) and 8 mM (×4) glucose, and viable counts performed through the growth cycle. The growth curve was unchanged by the increased glucose concentrations (Fig. 2). Friis suggested that the decline in viability when yellow colour shift was reached was due to the acidification of the medium. If so, this might be prevented by addition of alkali to the medium as the colour changes at the growth peak (60 h). Addition of alkali to neutralize the pH caused a marginal improvement in survival at 4–6 days although this difference was not marked (Fig. 2).

### 3.3 Solidifying Friis medium

In order to use Friis medium as the base for a solid medium to culture *M. hyopneumoniae*, different agar preparations were mixed with Friis base and or Mycoplasma Experience^®^ (ME) supplement and investigated for the ability to support the growth of *M. hyopneumoniae* colonies. The results are shown in Table 1.

Friis medium solidified with the commercially supplied ME agar grew colonies of *M. hyopneumoniae* 277/94 & 325/95. When Agar no.1, used widely in the solidification of bacteriological culture medium, was mixed with either Friis medium or ME supplement there was no growth of *M. hyopneumoniae*. Pre-treatment of the agar with activated charcoal, in an attempt to remove any growth-inhibiting components, did not alter the ability of medium made with either supplement to support *M. hyopneumoniae.* However, when either Purified agar or agarose were used as the solidifying agent, Friis medium, but not the ME supplement, grew colonies of *M. hyopneumoniae* (Table 1).

ME solid medium supported the growth of *M. hyorhinis*. When charcoal-treated agar, agarose or Purified agar were used, ME supplement did not grow *M. hyorhinis* colonies while Friis medium supported *M. hyorhinis* when solidified with each of the different agars.

**Fig. 3:**
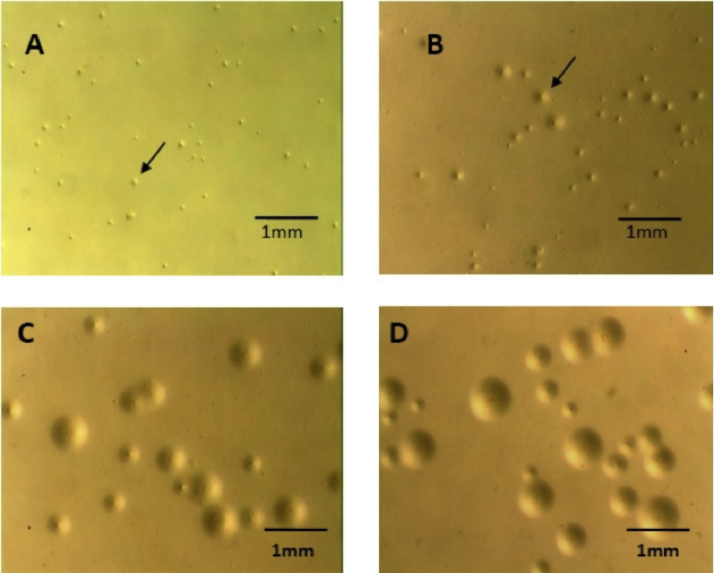
Colonies of *M. hyopneumoniae* 325 on different solid media preparations. Colonies were incubated for 7 days at 37 °C in a humidified box and photographed using a dissecting microscope. **A,** ME supplement solidified with ME agar; **B,** modified Friis medium solidified with 0.85% Purified agar; **C,** modified Friis medium solidified with 0.8% Purified agar and 0.1 mg/ml DEAE-dextran; **D,** modified Friis medium solidified with 0.9% agarose. Arrows indicate small *M. hyopneumoniae* colonies..

Based on the findings of Tauraso (1967) and as noted by Friis (1975) & Kobisch and Friis (1996), DEAE-dextran was added to the agar while molten and the growth of two strains of *M. hyopneumoniae* and *M. hyorhinis* were compared. Results are shown in Table 1. The inclusion of DEAE-dextran into Purified agar further enhanced the growth of colonies for strains of *M. hyopneumoniae* (strains 277/94 and 325/95) and appeared to be superior to the medium commercially available (Fig. 3).

### 3.4 Selective culture of *M. hyopneumoniae*

**Fig. 4:**
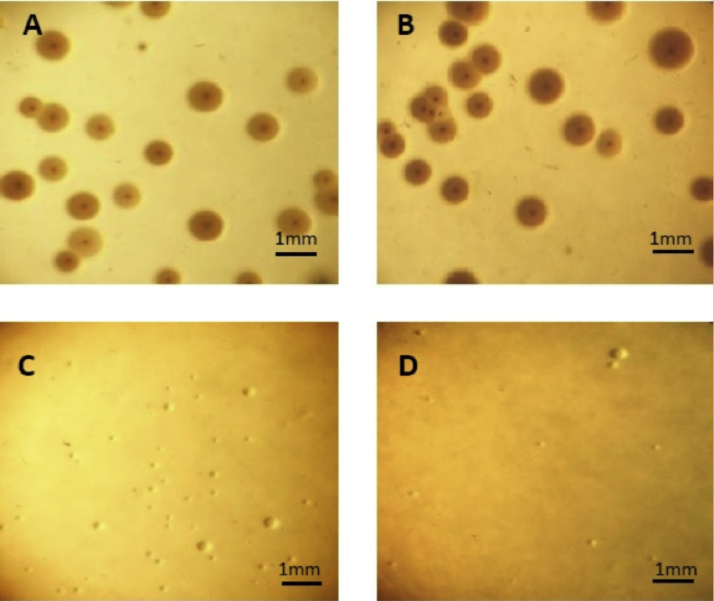
Suppression of *M. hyorhinis* growth following kanamycin selection on solid medium. Growth of a mixed mycoplasma culture containing 1:1 equal volumes of *M. hyopneumoniae* and *M. hyorhinis* mid-log phase culture, following 9 days incubation on Friis solid medium with and without 2 μg/ml kanamycin at 37 °C in a humidified box. **A,***M. hyopneumoniae* 277 and *M. hyorhinis*; **B,***M. hyopneumoniae* 325 and *M. hyorhinis*; **C,***M. hyopneumoniae* 277 and *M. hyorhinis* with 2 μg/ml kanamycin; **D,***M. hyopneumoniae* 277 and *M. hyorhinis* with 2 μg/ml kanamycin..

The susceptibility of *M. hyopneumoniae* 277/94 and 325/95, and *M. hyorhinis* Mhr1/09 was measured on solid medium containing four antimicrobials over a range of concentrations. The results are shown in Table 2. The MIC of kanamycin for *M. hyopneumoniae* isolates 325/95 and 277/94 was shown to be 8.0 μg/ml while *M. hyorhinis* appeared to be susceptible to a lower concentration, with an MIC of 1.0 μg/ml. Both strains of *M. hyopneumoniae* grew on medium containing 6.5 μg/ml. A mixed culture (equal numbers) of *M. hyopneumoniae* strain 277/94 and *M. hyorhinis* Mhr1/09 was used to assess the ability of kanamycin to selectively suppress the growth of *M. hyorhinis* on solid medium. After 8 days incubation in the absence of kanamycin, *M. hyorhinis* colonies outgrew *M. hyopneumoniae* as recognised by colony morphology (Fig. 4A & B). However, with addition of 2 μg/ml kanamycin only *M. hyopneumoniae* colonies appeared to grow (Fig. 4C & D).

The relative sensitivity of Mhr1/09 to kanamycin prompted the testing of more strains. A further 19 *M. hyopneumoniae* and 12 *M. hyorhinis* field isolates from the UK and Denmark, whose identity was confirmed by both PCR and growth characteristics, were tested for their susceptibility to kanamycin on MH selective medium (Table 3). All *M. hyopneumoniae* isolates grew in the presence of 2 μg/ml kanamycin. The isolates appeared to be unaffected by the incorporation of kanamycin into the medium with the exception of isolate Mp258/94 in which a small amount of growth suppression was noted in terms of colony size. The growth of all 12 *M. hyorhinis* isolates was completely inhibited by the addition of kanamycin in solid medium. Samples of pneumonic lung lesions were obtained from the abattoir over a period of 12 months. Cultures of these grown on MH selective medium were free from *M. hyorhinis* while approximately 60% of samples grown on non-selective Friis liquid and solid medium contained *M. hyorhinis* and became rapidly overgrown (Table 3).

## 4 Discussion

*M. hyopneumoniae* has been long recognised as difficult and unreliable to culture (Friis, 1971). Growth on solid medium has been considered particularly difficult with colonies growing to only 0.3–0.5 mm diameter in 7–10 days. This study was a systematic investigation to improve the medium for culture of this pathogen. The resulting improved medium was crucial in the successful development of transformation and transposon mutagenesis of *M. hyopneumoniae* as described by Maglennon et al., 2013a, Maglennon et al., 2013b.

Establishment of the growth curve for *M. hyopneumoniae* in Friis medium was a pre-requisite for studies to allow efficient transformation. Using viable counts on the solid medium described here, rather than colour change units or the indirect methods of colour shift (Friis, 1975) or measuring ATP by luminometry (Stemke and Robertson, 1990, Calus et al., 2010), the growth curve could be accurately determined.

It has been considered that glucose is the primary carbon and energy source for *M. hyopneumoniae.* Supplementation of the medium with additional glucose did not improve the viability or growth rate. This was consistent with the results of Stemke and Robertson (1990) who found no increase in growth rate or peak viability. They used 27 mM glucose. Since physiological glucose concentrations are normally between 3 and 5 mM, we considered an increase to 4 and 8 mM to be appropriate for the medium. Quenching of the acid formed in the medium using NaOH had only a minor effect on the viability, which by 8 days incubation had reached the level of the control without added NaOH. Thus it is likely that depletion of key nutrients rather than acid inhibition is a factor limiting growth in culture.

Initial experiments indicated that the purity of the agar used to solidify modified Friis medium greatly affected the growth of *M. hyopneumoniae*. Bacteriological agar was found to prevent the growth of *M. hyopneumoniae* colonies. Gould et al. (1944) had reported a similar inhibitory effect on the growth of *Neisseria gonorrhoeae* following the solidification of liquid medium with agar. Ley and Mueller (1946) identified a component, similar to a fatty acid, present in agar following methanol extraction. This extracted component present within agar was found to inhibit the growth of *N. gonorrhoeae* in the absence of starch. Activated charcoal is known to adsorb several impurities such as fatty acids. Therefore, attempts were made to remove potential inhibitory factors from the agar such as free fatty acid-like inhibitors by treatment with activated charcoal. Since activated charcoal had no effect on the agar, we conclude that this was failure to remove inhibitory factor(s).

When the commercial ME supplement was solidified with agar other than the supplied agar component it was unable to support the growth of either *M. hyorhinis* or *M. hyopneumoniae*. This suggested that an essential constituent for growth was absent from the liquid supplement and only present within the agar component. Solidifying Friis medium with Purified agar treated with DEAE-dextran confers a substantial improvement on the commercially available medium for growth of *M. hyopneumoniae*. Since DEAE is a positively charged group able to bind negatively charged ions, its action may be explained by chelation of the agaropectin present in agar which may be inhibitory to growth of *M. hyopneumoniae*. Agaropectin is the mixture of smaller molecules, comprising alternating residues of l- and d-galactose modified with acidic side-groups (particularly sulfate). This is consistent with the beneficial use of purified agarose, which is depleted of agaropectin, over agar no. 1 in this study. No equivalent improvement was seen from DEAE in culture of *M. hyorhinis*, suggesting that it is less sensitive to the acidic components of agaropectin.

Friis (1971) described a selective medium to suppress *M. hyorhinis*. This requires rabbit antiserum which is expensive and not always available for regular culture. In testing the relative sensitivity of *M. hyopneumoniae* and *M. hyorhinis* to four antimicrobials, our results showed that the latter was consistently more sensitive to kanamycin. Williams (1978) had previously shown that both *M. hyorhinis* and *M. hyopneumoniae* were susceptible to kanamycin (0.1 and 0.5 μg/ml respectively). However, different isolates were used, and determination of the MIC by Williams was in liquid medium. Nevertheless, incorporation of 2 μg/ml of kanamycin into the agar medium appeared to prevent the growth of *M. hyorhinis* following 8 days incubation. When mixed in equal volumes with *M. hyopneumoniae* strain 325, the *M. hyorhinis* did not form colonies while controls, without the addition of kanamycin, yielded only *M. hyorhinis* colonies from the mixed culture. Screening of *M. hyopneumoniae* and *M. hyorhinis* strains showed that this difference in kanamycin susceptibility was present between all *M. hyopneumoniae* and *M. hyorhinis* strains tested.

The addition of kanamycin can be used in place of the hyperimmune rabbit serum and gives a clean selection for the culture of *M. hyopneumoniae*, effectively preventing the growth of *M. hyorhinis* while allowing colonies of *M. hyopneumoniae* to form. The relative kanamycin resistance of *M. hyopneumoniae* has greatly improved the primary isolation of *M. hyopneumoniae* from abattoir tissue samples originating from 15 different farms. False negatives, resulting from *M. hyorhinis* overgrowth have been overcome using this selective medium.

## Conflicts of interest

None.

## Figures and Tables

**Table 1:** Growth of *M. hyopneumoniae* and *M. hyorhinis* on different solid media following 10 and 5 days incubation at 37 °C.

Sample	Liquid medium	Test agar	*M. hyopneumoniae*	*M. hyorhinis*
1	ME supplement	ME agar	+++	+++
2		1.3% Agar No. 1.	NG	NG
3		Charcoal-treated No. 1	NG	NG
4		1.3% agarose	NG	NG
5		1.3% Purified agar	NG	NG
6	Modified Friis (x2.8)	ME agar	+++	+++
7		1.3% Agar No. 1.	NG	+++
8		Charcoal-treated No. 1	NG	+++
9		1.3% agarose	+++	+++
10		1.3% Purified agar	++	+++

**Table 2:** MICs of antimicrobial agents for *M. hyopneumoniae* strain 277/94 and 325/95, and *M. hyorhinis* Mhr1/09 for kanamycin, tetracycline, chloramphenicol and puromycin determined on solid medium.

Antimicrobial agent	MIC μg/ml		
	277/94	325/95	Mhr1/09
Kanamycin	8.0	8.0	1.0
Tetracycline	0.125	0.125	0.06
Chloramphenicol	2.5	2.5	2.5
Puromycin	1.25	1.25	2.5

**Table 3:** Growth of *M. hyopneumoniae* and *M. hyorhinis* isolates on solid medium with and without 2 μg/ml kanamycin.

*M. hyopneumoniae* isolate	Source	Growth on solid medium	
		No kanamycin	2 μg/ml kanamycin
MH001	UK field isolates from lung lesions 2009–2010	+++	++
MH002		+++	+++
MH003		+++	+++
MH005		+++	+++
MH008		+++	+++
MH010		+++	+++
MH011		+++	+++
MH015		+++	++
MH016		+++	+++
MH031		+++	+++
MH033		+++	+++
MS2	Danish field isolates from lung lesions 1969	+++	+++
MS3		+++	+++
MS8		+++	+++
MS13		+++	+++
MS15		+++	+++
MS25		+++	+++
MS29		+++	+++
Mp258/94	Danish field isolates from lung lesions 1994–2007.	+++	+
Mp277/94		++	++
Mp473/95		+	+
Mp738/95		+++	+++
Mp325/95		++	++
Mp79/06		+++	++
Mp87/06		++	+
Mp92/06		+++	+++
Mp96//06		+++	+++
Mp85/07		+	+
Mp261/07		+++	+++

*M. hyorhinis* isolate	Source	Growth on solid medium	
		No kanamycin	2 μg/ml kanamycin
MHR01	UK field isolates from lung lesions 2009–2010.	+++	NG
MHR02		+++	NG
MHR04		+++	NG
MHR05		+++	NG
MHR06		+++	NG
MHR07		+++	NG
MHR08		+++	NG
MHR09		+++	NG
MHR10		+++	NG
MHR11		+++	NG
MHR12		+++	NG
P450		+++	NG

+++ large colonies; ++ medium colonies; + small colonies; NG = no colonies formed.
